# Hepatic glutamine synthetase controls *N*^5^-methylglutamine in homeostasis and cancer

**DOI:** 10.1038/s41589-022-01154-9

**Published:** 2022-10-24

**Authors:** Victor H. Villar, Maria Francesca Allega, Ruhi Deshmukh, Tobias Ackermann, Mark A. Nakasone, Johan Vande Voorde, Thomas M. Drake, Janina Oetjen, Algernon Bloom, Colin Nixon, Miryam Müller, Stephanie May, Ee Hong Tan, Lars Vereecke, Maude Jans, Gillian Blancke, Daniel J. Murphy, Danny T. Huang, David Y. Lewis, Thomas G. Bird, Owen J. Sansom, Karen Blyth, David Sumpton, Saverio Tardito

**Affiliations:** 1 Cancer Research UK Beatson Institute, Garscube Estate, Glasgow, UK; 2 Institute of Cancer Sciences, University of Glasgow, Glasgow, UK; 3 Department of Clinical Surgery, University of Edinburgh, Edinburgh, UK; 4 Bruker Daltonics GmbH & Co. KG, Bremen, Germany; 5 Host–Microbiota Interaction Lab, VIB Center for Inflammation Research, Ghent, Belgium; 6 Department of Internal Medicine and Pediatrics, Ghent University, Ghent, Belgium; 7 Centre for Inflammation Research, The Queen’s Medical Research Institute, University of Edinburgh, Edinburgh, UK

**Keywords:** Metabolomics, Metabolic pathways, Metabolism, Cancer

## Abstract

Glutamine synthetase (GS) activity is conserved from prokaryotes to humans, where the ATP-dependent production of glutamine from glutamate and ammonia is essential for neurotransmission and ammonia detoxification. Here, we show that mammalian GS uses glutamate and methylamine to produce a methylated glutamine analog, *N*^5^-methylglutamine. Untargeted metabolomics revealed that liver-specific GS deletion and its pharmacological inhibition in mice suppress hepatic and circulating levels of *N*^5^-methylglutamine. This alternative activity of GS was confirmed in human recombinant enzyme and cells, where a pathogenic mutation in the active site (R324C) promoted the synthesis of *N*^5^-methylglutamine over glutamine. *N*^5^-methylglutamine is detected in the circulation, and its levels are sustained by the microbiome, as demonstrated by using germ-free mice. Finally, we show that urine levels of *N*^5^-methylglutamine correlate with tumor burden and GS expression in a β-catenin-driven model of liver cancer, highlighting the translational potential of this uncharacterized metabolite.

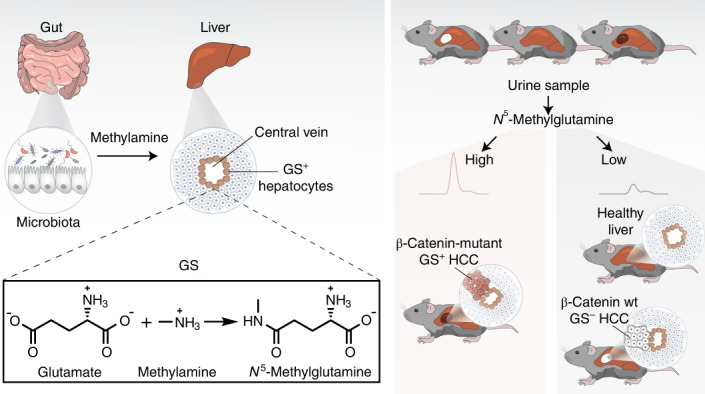

## Abstract (web summary)

Glutamine synthetase is the only enzyme that synthesizes glutamine in mammals. In vivo metabolomics showed that glutamine synthetase utilizes methylamine to produce *N*^5^-methylglutamine, whose levels correlate with tumor burden in a β+catenin+mutant liver cancer model.

## Main

Glutamine is a non-essential amino acid in mammals that is exclusively synthesized by the enzyme glutamine synthetase (GS)^1^. Despite having been isolated from mammalian tissue more than 70 years ago^2^, the pathophysiological role of GS has not been fully elucidated, and novel functions have recently been described in endothelium and macrophage biology^3,4^. Consistent with the role of glutamate and glutamine in neurotransmission^5^ and neurodevelopment^6^, inactivating point mutations in the GS active site cause severe neurodevelopmental defects that can cause perinatal death in humans^7^.

**Fig. 1:**
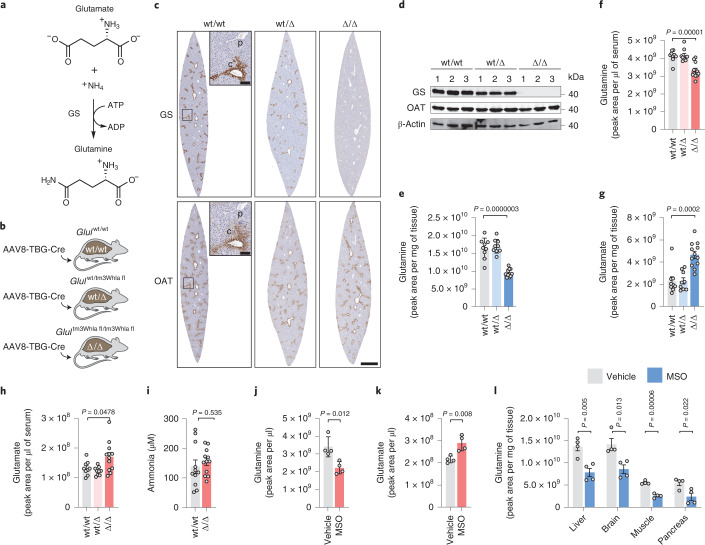
Effects of liver GS deficiency on glutamine metabolism. **a**, GS-catalyzed reaction. **b**, Administration of AAV8-TBG-Cre in mice with wt/wt, *Glul*^wt/tm3Whla fl^ and *Glul*^tm3Whla fl/tm3Whla fl^ genotypes results in mice with wt/wt, wt/Δ and Δ/Δ livers, respectively. **c**, Serial sections of mouse liver stained by IHC for GS and OAT, two markers of pericentral zones; scale bar, 1 mm. The insets show magnifications of the central vein (c) and portal vein (p); scale bar, 100 µm. The images shown are representative of three mice per genotype. **d**, Immunoblot of liver samples obtained from *n* = 3 mice per genotype. β-Actin is shown as a loading control. **e**,**f**, Glutamine levels in wt/wt (*n* = 9), wt/Δ (*n* = 9) and Δ/Δ (*n* = 12) livers (**e**) and sera (**f**) measured by LC–MS. **g**,**h**, Glutamate levels in the livers (**g**) and sera (**h**) of wt/wt (*n* = 9), wt/Δ (*n* = 9) and Δ/Δ (*n* = 12) mice. **i**, Ammonia concentration in blood samples from wt/wt (*n* = 11) and Δ/Δ (*n* = 12) mice. **j**,**k**, Glutamine (**j**) and glutamate (**k**) levels in the blood collected from mice 4 h after administration of vehicle (*n* = 4) and MSO (*n* = 4). **l**, Glutamine levels in the liver, brain, muscle and pancreas from mice treated as in **j** (*n* = 4 vehicle, *n* = 4 MSO). Data in **e**–**l** were analyzed by two-tailed Student’s *t*-test. Bars represent mean ± s.e.m., and each circle represents data from a single mouse. Source data

One of the many metabolic functions of the liver is to detoxify the ammonia released into the systemic circulation by metabolic processes. Liver failure causes an increase in the concentration of ammonia in blood circulation. While the mechanism of ammonia toxicity of the central nervous system is not fully understood, the clinical manifestations of severe hyperammonemia include impaired brain functions, known as hepatic encephalopathy, that can lead to brain injury and death^8^. Hepatic GS is selectively expressed in hepatocytes surrounding the central veins, where residual ammonia that has not been detoxified by the urea cycle is incorporated into the amidic group of glutamine by an ATP-dependent reaction that uses glutamate as cosubstrate^8,9^ (Fig. 1a). Hence, the role of GS in physiopathology has been studied both in relation to its ammonia-clearing and glutamine-producing activities^4,8–11^.

We applied orthogonal pharmacological and genetic approaches in mouse and cellular models to show that the metabolic effects of GS extend beyond the regulation of its canonical substrates and product and connect microbiome and hepatic metabolism by synthesizing *N*^5^-methylglutamine.

## Results

### Metabolic effects of hepatic GS deletion

**Extended Data Fig. 1:**
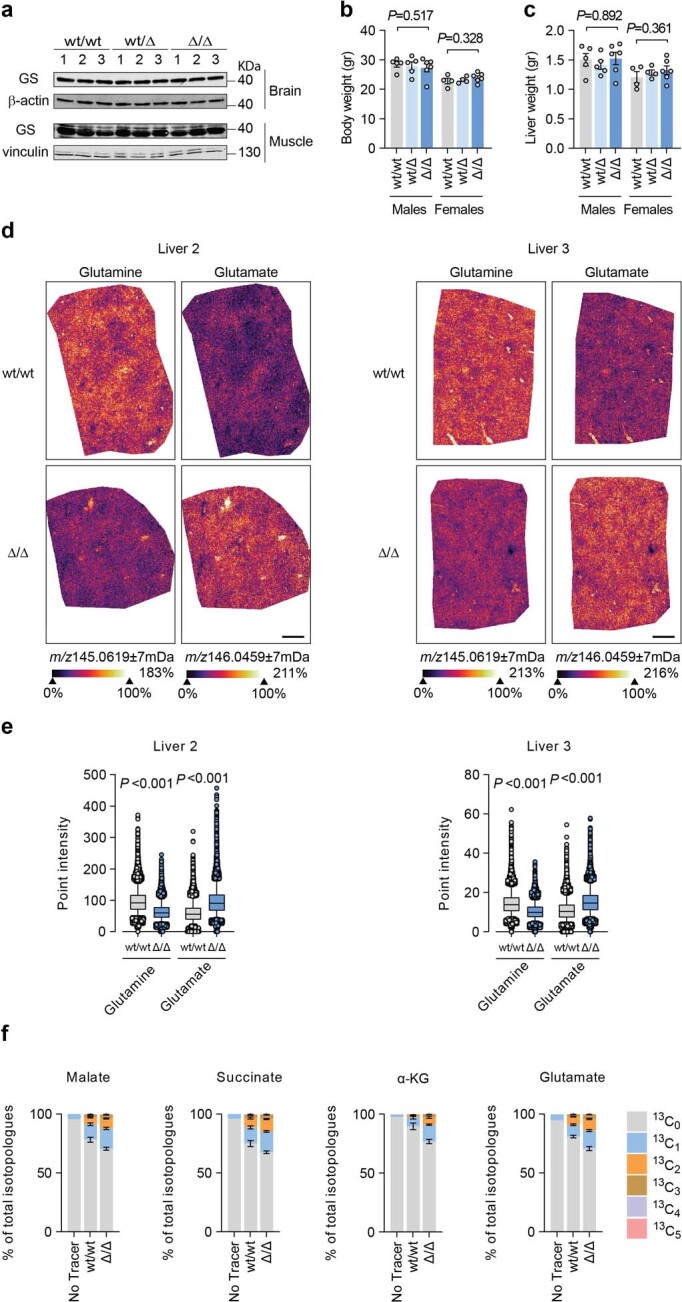
Metabolic effects of hepatic GS deletion. **a**, Immunoblot for GS of brain (top) and muscle (bottom) samples from wt/wt (n = 3), wt/Δ (n = 3), Δ/Δ (n = 3) mice shown in Fig. 1d. β-actin and vinculin are shown as loading controls. **b**, Body weights and (**c**) liver weights of male wt/wt (n = 5), wt/Δ (n = 5), Δ/Δ (n = 6) and female wt/wt (n = 4), wt/Δ (n = 4), Δ/Δ (n = 6) mice. Two-tailed Student’s t-test. Bars are mean ± s.e.m. **d**, Glutamine and glutamate relative abundance is shown in sections of frozen liver samples from wt/wt (n = 1 male, n = 1 female) and Δ/Δ mice (n = 1 male, n = 1 female) analyzed with mass spectrometry metabolic imaging. Data were normalized with root mean square method. Scale bar = 1 mm. **e**, Glutamine and glutamate quantification of the metabolic images presented in (d) (n = 2 wt/wt, n = 2 Δ/Δ). Each box and whiskers represents data from one image obtained from one liver. Box with bounds at 25^th^-75^th^ percentiles, line at median, and whiskers at 5^th^-95^th^ percentiles, each data point represents the relative intensity of one pixel. Two-tailed Student’s t-test. **f**, Relative levels of ^13^C isotopologues for the indicated metabolites in the liver extracts of a no tracer control mouse (n = 1) and in wt/wt (n = 4) and Δ/Δ (n = 5) mice administered with U-^13^C_6_-glucose. Bars are mean ± s.e.m. Source data

The deletion of *Glul* in the hepatocytes of adult mice carrying the *Glul*^tm3Whla^ floxed allele (*Glul*^tm3Whla fl^)^8^ was achieved by administering adeno-associated virus with TBG promoter-driven expression of Cre (AAV8-TBG-Cre; Fig. 1b)^12,13^. While heterozygous deletion of hepatic *Glul* (wild type/Δ (wt/Δ)) caused an ~40% decrease in GS expression (Fig. 1c,d), homozygous deletion (Δ/Δ) resulted in complete liver-specific loss of GS (Fig. 1c,d and Extended Data Fig. 1a), without affecting the expression of ornithine aminotransferase (OAT), another metabolic marker of the pericentral zone (Fig. 1c,d). Hereafter, mice with liver-specific recombined *Glul*^tm3Whla fl^ alleles are referred to as wt/wt, wt/Δ and Δ/Δ. GS deletion did not affect body or liver weight (Extended Data Fig. 1b,c), and, in accordance with human GS deficiency syndrome, the heterozygous deletion of GS did not result in appreciable metabolic changes (Fig. 1e–h)^14^. Despite GS expression being limited to only ~7% of all hepatocytes, its homozygous deletion caused an ~40% decrease in glutamine levels in the liver (Fig. 1e) and an ~20% decrease in systemic blood circulation (Fig. 1f). Whereas circulating ammonia levels were not significantly altered (Fig. 1i), glutamate levels were elevated in Δ/Δ liver and serum (Fig. 1g,h). Comparable results for the circulating levels of glutamine and glutamate were obtained after treatment with methionine sulfoximine (MSO; Fig. 1j,k), an irreversible GS inhibitor that decreased glutamine levels in GS-expressing organs, including the liver, brain, muscle and pancreas (Fig. 1l).

**Fig. 2:**
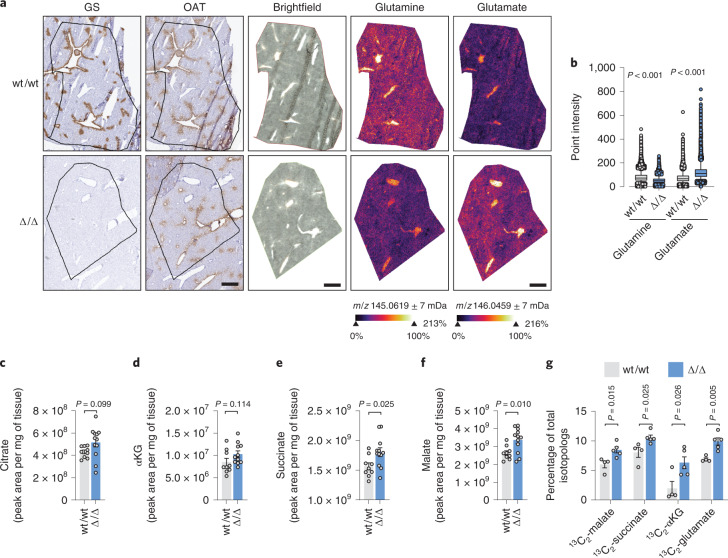
Metabolic imaging and glucose tracing in GS-deficient liver. **a**, Serial sections of frozen liver samples from wt/wt and Δ/Δ female mice. Left, IHC staining for GS and OAT. Right, MS metabolic imaging of glutamine and glutamate. Data were normalized with the root mean square method; scale bar, 1 mm. Images are representative of *n* = 3 livers per genotype (Extended Data Fig. 1d,e). **b**, Quantification of glutamine and glutamate in the regions of interest shown in **a** (*n* = 1 wt/wt, *n* = 1 Δ/Δ). Boxes have bounds at the 25th to 75th percentiles, the lines represent the medians and whiskers show the 5th to 95th percentiles; each data point represents the relative intensity of one pixel. Data were analzyed by two-tailed Student’s *t*-test. **c**–**f**, Levels of citrate (**c**), α-ketoglutarate (αKG; **d**), succinate (**e**) and malate (**f**) in the livers of wt/wt (*n* = 9) and Δ/Δ (*n* = 12) mice. **g**, Relative levels of ^13^C_2_ isotopolog in the livers of wt/wt (*n* = 4) and Δ/Δ (*n* = 5) mice administered U-^13^C_6_-glucose. Data were analyzed by two-tailed Student’s *t*-test. Bars represent mean ± s.e.m., and each circle represents data from a single mouse. Source data

To determine if the distinctive zonation of GS expression in the liver could affect the levels of glutamine and glutamate in different metabolic zones, we applied mass spectrometry (MS)-based metabolic imaging. In wt/wt liver, glutamine and glutamate levels were comparable between pericentral (GS^+^OAT^+^) and periportal (GS^−^OAT^−^) areas (Fig. 2a). Consistently, GS deletion decreased glutamine levels and increased glutamate levels throughout the liver tissue (Fig. 2a,b and Extended Data Fig. 1d,e), suggesting that GS activity determines glutamine and glutamate concentrations across the different metabolic zones of the liver lobule.

The ~30% increase in circulating levels of glutamate observed in Δ/Δ mice (Fig. 1h) demonstrates that hepatic glutamine synthesis regulates systemic glutamate metabolism^15^. However, the increased hepatic levels of tricarboxylic acid (TCA) cycle intermediates observed in Δ/Δ liver (Fig. 2c–f) are suggestive of a decreased flux draining from the TCA to glutamine (that is, cataplerotic) imposed by the lack of glutamine synthesis. To directly investigate this hypothesis, we traced ^13^C_6_-glucose in wt/wt and Δ/Δ mice. The results showed that the enrichment in glucose-derived carbons of malate, succinate, α-ketoglutarate and glutamate is higher in Δ/Δ than in wt/wt livers (Fig. 2g and Extended Data Fig. 1f). Together, these results demonstrate that the hepatic synthesis of glutamine is fueled at least in part by in situ glutamate production from glucose.

### In vivo levels of *N*^5^-methylglutamine are GS dependent

**Fig. 3:**
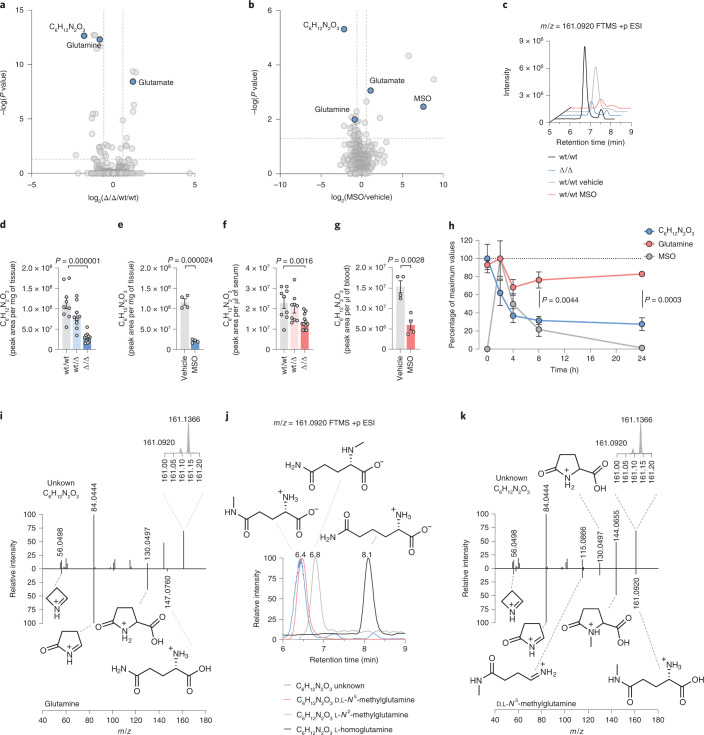
Untargeted metabolomics reveals *N*^5^-methylglutamine as a GS product. **a**,**b**, Manually curated volcano plots obtained from an untargeted metabolomic analysis of livers from wt/wt (*n* = 9) and Δ/Δ (*n* = 12) mice (**a**) or from mice treated with vehicle (*n* = 4) or MSO (*n* = 4) (**b**). Data were analyzed by analysis of variance and Tukey honest significant difference post hoc test. **c**, Extracted ion chromatograms of one representative liver sample from mice described in **a** and **b**. FTMS +p ESI, Fourier transform mass spectometry in positive polarity coupled with electrospray ionisation. **d**,**e**, Levels of the compound with formula C_6_H_12_N_2_O_3_ in the livers of wt/wt (*n* = 9), wt/Δ (*n* = 9) and Δ/Δ (*n* = 12) mice (**d**) or in the livers collected from mice 4 h after administration of vehicle (*n* = 4) or MSO (*n* = 4) (**e**). **f**,**g**, Levels of the compound with formula C_6_H_12_N_2_O_3_ in the sera (**f**) or blood (**g**) of mice described in **d** and **e**. Data in **d**–**g** were analyzed by two-tailed Student’s *t*-test. **h**, Relative levels of the compound with formula C_6_H_12_N_2_O_3_ in the blood of vehicle-treated (*n* = 4) or MSO-treated (*n* = 4) mice. MSO was administered immediately after the first blood collection at 0 h. For each metabolite, the percentage of the maximal peak area values averaged at each time point is reported. Data were analyzed by two-tailed Student’s *t*-test. Circles represent mean ± s.e.m. (*n* = 4). **i**, Mirror plot of MS/MS spectra from wt/wt liver samples (*n* = 2) comparing parent and fragment ions generated from the compound with formula C_6_H_12_N_2_O_3_ (top) to glutamine (bottom). The inset shows a magnified *m*/*z* range of 161.0 to 161.2 showing the ion of interest (*m*/*z* 161.0920) and a coisolated ion (*m*/*z* 161.1366). The structures of putative glutamine fragments are shown (Extended Data Fig. 2b). **j**, Overlaid extracted ion chromatograms of *m*/*z* 161.0920 obtained from a wt/wt liver sample (*n* = 1, C_6_H_12_N_2_O_3_ unknown), d,l-*N*^5^-methylglutamine standard, l-*N*^2^-methylglutamine standard and l-homoglutamine standard. The retention times and molecular structures of these compounds are shown. **k**, Mirror plot of MS/MS data from a wt/wt liver sample (*n* = 1) comparing the compound with formula C_6_H_12_N_2_O_3_ (top) to d,l-*N*^5^-methylglutamine standard (bottom). The proposed structures of parent and fragment ions derived from d,l-*N*^5^-methylglutamine are shown (Extended Data Fig. 2c). Source data

To broaden our understanding of the role of GS in liver metabolism, we performed a liquid chromatography–MS (LC–MS)-based untargeted analysis on Δ/Δ livers and on livers from mice treated with MSO. The comparisons with the respective controls revealed an unexpected feature with a predicted molecular weight of 160.08479 ± 1.2 ppm and a molecular formula of C_6_H_12_N_2_O_3_ that was significantly downregulated after GS deletion or pharmacological inhibition (Fig. 3a,b). The corresponding extracted ion chromatograms revealed a peak with lower intensity in Δ/Δ and MSO-treated livers than in respective controls (Fig. 3c). Quantification of the normalized peak areas showed decreases of ~75% and ~80% in Δ/Δ and MSO-treated livers, respectively, compared to controls (Fig. 3d,e). C_6_H_12_N_2_O_3_ levels were also decreased in the sera of Δ/Δ and MSO-treated mice (~60% and ~50%, respectively; Fig. 3f,g). Circulating levels of C_6_H_12_N_2_O_3_, glutamine and MSO were monitored for 24 h after administration of MSO (Fig. 3h). MSO levels reached a maximum of 18.6 ± 7.2 μM (mean ± s.d.; *n* = 4) 2 h after administration and decreased to 0 at 24 h. Glutamine levels transiently dropped between 2 and 4 h but recovered thereafter. Conversely, the levels of C_6_H_12_N_2_O_3_ progressively diminished after MSO administration, reaching ~27% of the initial levels at 24 h. These results demonstrate that the circulating levels of glutamine and C_6_H_12_N_2_O_3_ respond to irreversible GS inhibition with different kinetics, suggesting that alternative sources of glutamine (for example, diet or autophagy) may compensate for the lack of glutamine synthesis, while C_6_H_12_N_2_O_3_ production strictly depends on GS activity.

**Extended Data Fig. 2:**
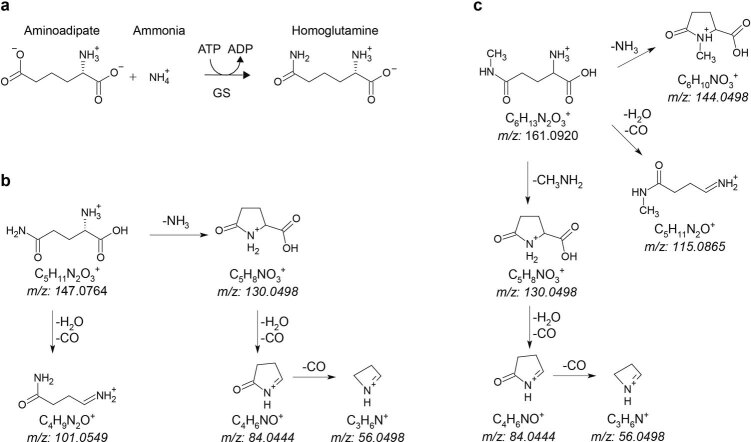
Proposed mechanisms of reaction and fragmentation. **a**, Schematic of the proposed reaction where α-aminoadipic acid and ammonia are used by GS to produce homoglutamine^16^. **b**, Proposed mechanism for glutamine fragmentation^17^. **c**, Proposed mechanism for N5-methylglutamine fragmentation.

To identify the compound with formula C_6_H_12_N_2_O_3_, we analyzed wt/wt livers using tandem mass spectrometry (MS/MS). The comparison of the fragmentation patterns of glutamine (C_5_H_10_N_2_O_3_) and C_6_H_12_N_2_O_3_ revealed the presence of three common fragments (*m*/*z* 56.0498, 84.0444 and 130.0497; Fig. 3i), supporting the hypothesis of C_6_H_12_N_2_O_3_ being a glutamine analog with an additional methylene or methyl group. The addition of a methylene group to the glutamine carbon backbone could lead to l-homoglutamine, reported on one occasion to be synthesized by mammalian GS from α-aminoadipic acid and ammonia (Extended Data Fig. 2a)^16^. However, the analytical standard for l-homoglutamine was chromatographically separated from the unknown compound present in liver extracts, refuting this hypothesis (Fig. 3j). Next, we tested the chromatographic response of two glutamine analogs with the methyl group bound to either the aminic (*N*^2^-methylglutamine) or amidic nitrogen (*N*^5^-methylglutamine). The two compounds were chromatographically separated, and the retention time of *N*^5^-methylglutamine coincided with that of the unknown compound present in the liver samples (Fig. 3j). Further MS/MS analysis showed matching fragmentation patterns for the analytical standard and the unknown compound (common fragments *m*/*z* 56.0498, 84.0444, 115.0866, 130.0497 and 144.0655), identifying it as *N*^5^-methylglutamine (Fig. 3k; proposed mechanisms of fragmentation for glutamine^17^ and *N*^5^-methylglutamine are shown in Extended Data Fig. 2b,c).

### Human GS synthesizes *N*^5^-methylglutamine in vitro

**Fig. 4:**
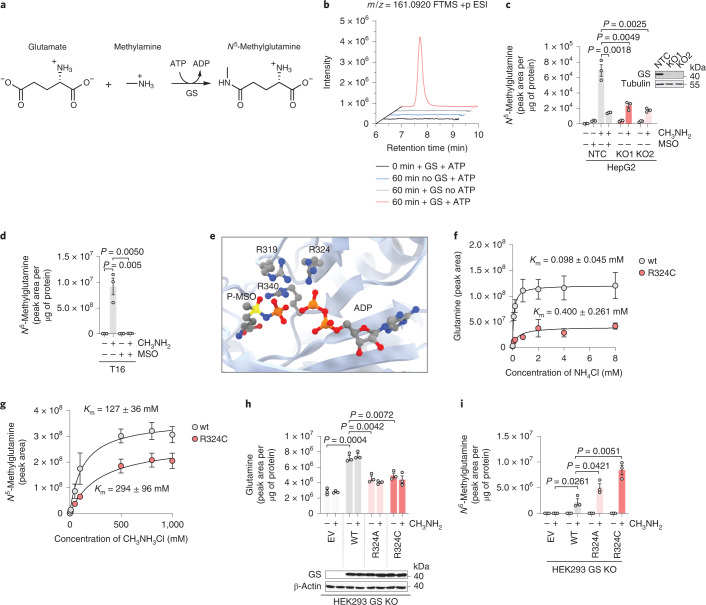
GS synthesizes *N*^5^-methylglutamine from glutamate and methylamine. **a**, Non-canonical reaction catalyzed by GS. **b**, Reaction mixtures described in the Methods and modified as indicated in the figure were sampled at 0 and 60 min from the addition of 40 mM methylamine. Representative extracted ion chromatograms for *m*/*z* 161.0920 are shown. **c**, HepG2 cells deficient for GS (KO1 and KO2) or non-targeting control (NTC) were grown in glutamine-free medium for 24 h and incubated with methylamine (CH_3_NH_2_, 0.8 mM) or MSO (1 mM) for an additional 24 h. Data were analyzed by two-tailed Student’s *t*-test. Bars represent mean ± s.e.m., and each data point represents an independent experiment (*n* = 3). The inset shows immunoblotting analysis of GS. Tubulin is shown as a loading control (*n* = 1). **d**, T16 cells were incubated with methylamine (0.4 mM) or MSO (1 mM) as indicated for 48 h. Data were analyzed by two-tailed Student’s *t*-test. Bars represent mean ± s.e.m., and each data point represents an independent experiment (*n* = 3). **e**, The active site of human GS with highlighted arginine residues, phospho-MSO (P-MSO) and ADP (PDB 2QC8 visualized with UCSF Chimera v1.15). **f**,**g**, GS wt and mutant proteins were purified as described in the Methods and in Extended Data Fig. 3c–f. *K*_m_ values for ammonia (**f**) and methylamine (**g**) were calculated using the Michaelis–Menten equation. Samples were collected from the reaction mixtures 15 min after methylamine or ammonia addition. Data points represent mean ± s.e.m. of *n* = 3 independent experiments. **h**,**i**, HEK293 cells deleted for GS (GS KO) were transfected with an empty vector (EV) control or vectors encoding GS wt or R324A and R324C mutant variants. Twenty-four hours after transfection, cells were incubated with methylamine (0.4 mM) for an additional 24 h, and glutamine (**h**), GS (**h** immunoblot inset; β-actin is shown as a loading control; *n* = 1) and *N*^5^-methylglutamine (**i**) levels were measured. Data were analyzed by two-tailed Student’s *t*-test. Bars represent mean ± s.e.m., and each data point represents an independent experiment (*n* = 3). Source data

To our knowledge, the biosynthesis of *N*^5^-methylglutamine has not been reported in mammals, and in bacteria, *N*^5^-methylglutamine can be obtained by the transfer of a methyl group from a methyl donor, such as *S*-adenosyl-l-methionine, as described for peptide-bound glutamine^18^, or by the nucleophilic attack of methylamine to the γ-carboxylic group of glutamate (Fig. 4a)^19,20^.

**Extended Data Fig. 3:**
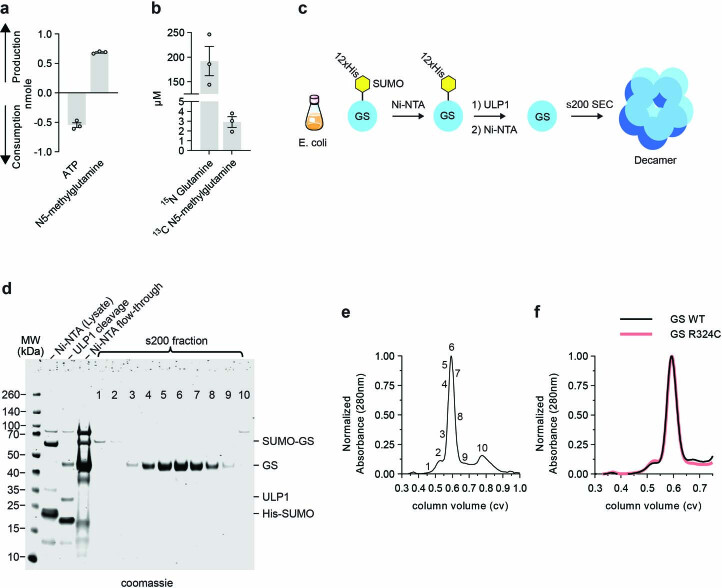
Stoichiometry of GS activities and purification of GS wild type and R324C mutant proteins. **a**, Recombinant human GS protein was incubated in 50 μl of the reaction mixture described in the methods section with 40 mM methylamine. The differences in nanomoles of ATP and N5-methylglutamine between samples harvested before and 60 minutes after methylamine addition are shown. Each data point represents an independent experiment (n = 3). Bars are mean ± s.e.m.. **b**, Recombinant human GS protein was incubated in 500 μl of the reaction mixture described in the Methods section. The reaction was started by adding a solution containing 20 mM ^13^C methylamine and 20 mM ^15^N ammonia. ^13^C N5-methylglutamine and ^15^N glutamine were quantified in samples harvested 60 minutes after the reaction start. Each data point represents an independent experiment (n = 3). Bars are mean ± s.e.m.. **c**, Schematic of the purification steps for GS WT and GS R324C described in the Methods section. **d**, Coomassie stained SDS^−^PAGE gel showing the purification steps and size exclusion fractions for GS WT protein. SDS-PAGE gels for the purification of GS WT and GS R324C showed comparable results (n = 1). **e**, Superdex 200 size exclusion profile for the GS WT protein. **f**, Overlay of size exclusion profiles for GS WT and GS R324C loaded separately onto a Superdex 200 column. The elution profiles were overlaid demonstrating an identical oligomeric state for GS WT and mutant. Source data

To demonstrate that GS is the source of *N*^5^-methylglutamine and test if methylamine is used as substrate, we used purified human recombinant GS. The LC–MS analysis of the reaction mixture containing methylamine sampled at the reaction start and after 60 min demonstrated that GS production of *N*^5^-methylglutamine is ATP dependent (Fig. 4b and Extended Data Fig. 3a). Moreover, if equimolar amounts of ammonia and methylamine were added to the recombinant GS, *N*^5^-methylglutamine and glutamine were synthesized with a stoichiometric ratio of 1:65 (Extended Data Fig. 3b).

Next, we tested if human cells could produce *N*^5^-methylglutamine. GS expression is high in liver and brain, and we found that cancer cell lines derived from these organs (HepG2 and T16 (ref. ^21^)) produce *N*^5^-methylglutamine after supplementation of methylamine (Fig. 4c,d), whereas Cas9-mediated deletion of GS in HepG2 cells significantly inhibited *N*^5^-methylglutamine production (Fig. 4c). Comparable results were obtained by inhibiting GS with MSO treatment in both HepG2 and T16 cell lines (Fig. 4c,d). To assess the relevance of the newly described activity of GS to human pathology, we investigated pathogenic mutations affecting the GS active site (Fig. 4e), which cause a rare inborn error of metabolism called GS deficiency^14^. First, we purified a human recombinant GS with the R324C mutation (Extended Data Fig. 3c–f) and found that this mutation decreased the affinity for ammonia (Fig. 4f) more than that for methylamine (Fig. 4g). Subsequently, we tested the effects of pathogenic mutations in HEK293 cells where the endogenous *GLUL* alleles were deleted (GS KO), and wt GS or the R324C and R324A mutants were reexpressed. In cells expressing R324C and R324A mutants, glutamine levels were reduced by half compared to cells expressing wt GS (Fig. 4h), in line with the decreased glutamine levels found in individuals with GS mutations^14^. Conversely, cellular levels of *N*^5^-methylglutamine were markedly increased by the mutant variants compared to the wt control (Fig. 4i). These observations indicate that these mutations disproportionately affect the catalytic activity of GS with high affinity for ammonia (approximately fourfold; Fig. 4f), conferring a competitive advantage to the low-affinity substrate methylamine (Fig. 4g). Altogether, these results demonstrate that human GS synthesizes *N*^5^-methylglutamine from glutamate and methylamine and show that this alternative activity is favored by human pathogenic mutations occurring at the active site of the enzyme.

### Methylamine is a GS substrate in vivo

**Fig. 5:**
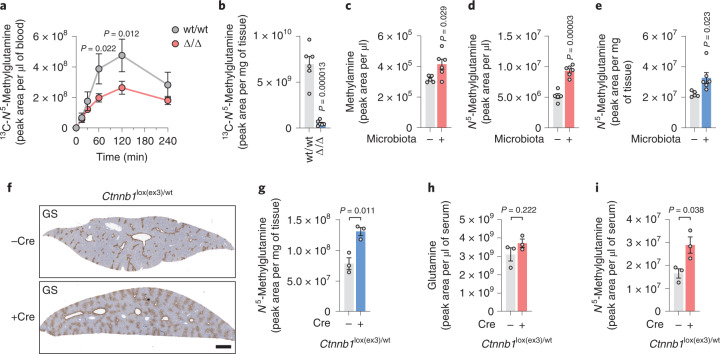
The microbiome and β-catenin activation increase *N*^5^-methylglutamine synthesis. **a**, ^13^C-*N*^5^-methylglutamine levels in the blood of wt/wt (*n* = 3) and Δ/Δ (*n* = 3) mice immediately before and 15, 30, 60, 120 and 240 min after injection of ^13^C-methylamine (2 mmol per kg body weight). *P* values refer to the comparison of wt/wt to Δ/Δ levels at 60 and 120 min. **b**, ^13^C-*N*^5^-methylglutamine levels in the livers of wt/wt (*n* = 6) and Δ/Δ (*n* = 6) mice 2 h after ^13^C-methylamine administration. **c**–**e**, Methylamine (**c**) and *N*^5^-methylglutamine (**d**) levels in the sera and *N*^5^-methylglutamine levels in the liver tissue (**e**) of female germ-free mice administered with vehicle control (*n* = 5) or with SPF microbiota (*n* = 6). **f**, Representative images of IHC staining for GS in livers of *Cnntb1*^fl(ex3)/wt^ male mice 4 d after administration of AAV8-Null-Cre or AAV8-TBG-Cre; scale bar, 1 mm. **g**, Levels of *N*^5^-methylglutamine in the livers of AAV8-Null-Cre (*n* = 3) or AAV8-TBG-Cre (*n* = 3) mice described in **f**. **h**,**i**, Glutamine (**h**) and *N*^5^-methylglutamine (**i**) levels in the sera of the mice (*n* = 3) described in **f**. Data in **a**–**e** and **g**–**i** were analyzed by two-tailed Student’s *t*-test. Bars represent mean ± s.e.m. Source data

**Extended Data Fig. 4:**
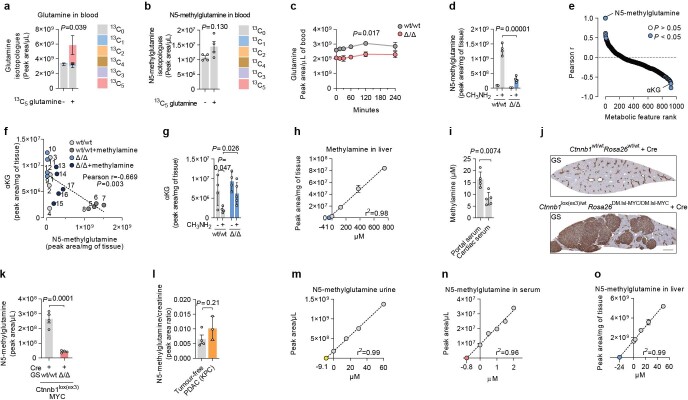
N5-methylglutamine *in vivo* metabolic origin, its effects on liver metabolism, and its levels in murine cancer models. **a**, **b**, Glutamine and N5-methylglutamine isotopologue levels in the blood of wt/wt mice (n = 4, 2 females and 2 males) before (-) and after (+) administration of ^13^C_5_ glutamine. *P* values refer to the sum of all detected isotopologues. Two-tailed ratio paired Student’s t-test. Bars are mean ± s.e.m.. **c**, Levels of glutamine in the blood of wt/wt (n = 3) and Δ/Δ (n = 3) mice injected with ^13^C methylamine. *P* value refers to the comparison of wt/wt to Δ/Δ blood at 120 minutes. Two-tailed Student’s t-test. Circles are mean ± s.e.m.. **d**, N5-methylglutamine levels in the liver tissue of wt/wt (n = 4, 3 females and 1 male) and Δ/Δ (n = 5, 3 females and 2 males) mice administered 0.1% w/v methylamine, supplemented to drinking water for 5 months, or in wt/wt (n = 4, 2 females and 2 males) and Δ/Δ (n = 4, 3 females and 1 male) control mice. Two-tailed Student’s t-test. Bars are mean ± s.e.m.. **e**, Metabolic features identified by untargeted metabolomic analysis of liver tissue of the mice described in (d) and ranked according to their correlation values with N5-methylglutamine levels shown in (d) (n = 4 wt/wt and n = 5 Δ/Δ administered methylamine, n = 4 wt/wt and n = 4 Δ/Δ with water control). Cut-off *P* values refers to a two tailed Pearson correlation analysis (n = 17). **f**, Two tailed Pearson correlation analysis (n = 17) between the α-ketoglutarate and N5-methylglutamine in the liver tissues of mice described in (d). **g**, α-ketoglutarate levels in liver tissue of the mice described in (d). n = 4 wt/wt and n = 5 Δ/Δ administered methylamine, n = 4 wt/wt and n = 4 Δ/Δ with water control. Two-tailed Student’s t-test. Bars are mean ± s.e.m.. **h**, Methylamine concentrations quantified with a standard addition method in liver tissue (n = 1) assuming that 1 g of tissue = 1 ml. Circles are mean ± s.e.m. of 2 technical replicates. **i**, Methylamine concentrations in portal and cardiac sera. Wild type C57Bl6/J mice were fasted overnight for 16 hours and refed for 4 hours (post-absorptive state) before being anesthetized. For each mouse samples of blood were collected by venipuncture from the portal vein and subsequently by cardiac puncture (n = 5 mice). Two-tailed ratio paired Student’s t-test. Data are mean ± s.e.m.. **j**, Images of IHC staining for GS in liver sections from *Ctnnb1*^wt/wt^, *Rosa26*^wt/wt^ or *Ctnnb1*^lox(ex3)/wt^, *Rosa26*^DM.lsl-MYC/DM.lsl-MYC^ female mice, harvested 107 and 151 days after administration of AAV8-TBG-Cre. Scale bar = 1 mm. The images shown are representative of 3 mice per genotype. **k**, Levels of N5-methylglutamine in the serum sampled at clinical endpoint from tumour-bearing *Ctnnb1*^lox(ex3)/wt^, *Rosa26*^DM.lsl-MYC/DM.lsl-MYC^ male mice with GS wt/wt (n = 4) or GS Δ/Δ (n = 4), described in Fig. 6e. Two-tailed Student’s t-test. Bars are mean ± s.e.m.. **l**, Normalized urine levels of N5-methylglutamine from tumour-free mice described in Fig. 6e (n = 4) and from K-ras^LSL.G12D/+^, Trp53^R172H/+^, Pdx-1-Cre (KPC) mice with pancreatic ductal adenocarcinoma (PDAC) sampled at clinical endpoint (n = 3). Two-tailed Student’s t-test. Bars are mean ± s.e.m.. **m**-**o**, N5-methylglutamine concentrations quantified with a standard addition method in urine (m) (n = 1, pooled urine from 5 mice), serum (n) (n = 1 pooled sera from 4 mice), and liver tissue (o) (assuming that 1 g of tissue =1 ml, n = 1). Coloured circles indicate the x intercept, and their absolute values correspond to the μM concentrations of metabolites in the samples without standard addition. Circles are mean ± s.e.m. of 2 (m, o) or 3 (n) technical replicates. Source data

To test if *N*^5^-methylglutamine could also be produced by methylation of glutamine, we traced ^13^C_5_-glutamine in wt/wt mice. Although ^13^C_5_-glutamine levels in circulation were significantly increased, ^13^C_5_-*N*^5^-methylglutamine remained undetectable (Extended Data Fig. 4a,b), strengthening the in vivo validity of the working model where *N*^5^-methylglutamine is synthesized by GS from glutamate and methylamine. To investigate whether systemic methylamine could modulate the production of *N*^5^-methylglutamine, we injected ^13^C-methylamine into wt/wt and Δ/Δ mice. The administration of ^13^C-methylamine did not significantly perturb circulating glutamine levels (Extended Data Fig. 4c). By contrast, ^13^C-*N*^5^-methylglutamine levels peaked at 2 h after ^13^C-methylamine injection, at which point the levels reached in Δ/Δ mice were significantly lower than those in wt/wt mice (Fig. 5a). Consistently, the levels of ^13^C-*N*^5^-methylglutamine in the liver were approximately tenfold lower in Δ/Δ mice than in wt/wt mice (Fig. 5b). This demonstrates that *N*^5^-methylglutamine levels are modulated in vivo by circulating methylamine via hepatic GS activity. Similarly, *N*^5^-methylglutamine levels were markedly decreased (approximately fivefold) in the Δ/Δ liver compared to wt/wt in mice supplemented for 5 months with 0.1% (wt/vol) methylamine in the drinking water (Extended Data Fig. 4d). Next, we performed untargeted metabolomics on liver tissue samples from wt/wt and Δ/Δ mice untreated or chronically supplemented with methylamine. To identify the metabolic effects specific for the liver-produced *N*^5^-methylglutamine, we ranked the metabolic features based on their Pearson correlation coefficients with *N*^5^-methylglutamine levels (Extended Data Fig. 4e). The ranking identified α-ketoglutarate as the metabolic feature with the second most stringent anticorrelation with *N*^5^-methylglutamine (Pearson *r* = −0.67 and *P* = 0.003; Extended Data Fig. 4e,f). In fact, α-ketoglutarate levels were significantly decreased by methylamine in wt/wt but not in Δ/Δ livers (Extended Data Fig. 4g), suggesting that *N*^5^-methylglutamine could negatively regulate hepatic levels of α-ketoglutarate.

### *N*^5^-methylglutamine synthesis is sustained by the microbiome

It has been shown that methylamine can be produced by the human enzyme PAD4 during the demethylimination of histone methyl-arginine^22^. Moreover, the levels of methylamine excreted in urine increase after sarcosine administration in rats^23^ and choline ingestion in humans^24^. However, the metabolic origin of methylamine in liver tissue (~41 μM; Extended Data Fig. 4h) is not fully understood. In analogy to the production of trimethylamine, which largely depends on intestinal microbiome metabolism^25^, we tested whether methylamine production could also be microbiota dependent. In line with this hypothesis, the concentration of methylamine in serum sampled from the portal vein was twofold higher (16 μM) than the concentration found in the systemic circulation (Extended Data Fig. 4i). To further investigate the role of the microbiome, age-matched germ-free mice were orally administered either sterile PBS or a solution containing the microbiota derived from a specific pathogen-free (SPF) donor. The sera obtained from microbiome-reconstituted mice showed that methylamine levels were significantly increased compared to germ-free mice (Fig. 5c). Consequently, the levels of *N*^5^-methylglutamine increased by ~50% in sera and by ~30% in liver tissue of mice with an active microbiome (Fig. 5d,e). These results demonstrate that hepatic GS mediates the conversion of microbiome-derived methylamine to *N*^5^-methylglutamine.

### Liver β-catenin mutation drives *N*^5^-methylglutamine synthesis

It has been shown that *GLUL* is a direct transcriptional target of β-catenin in the liver^26^. The activation of the WNT/β-catenin signaling pathway drives human liver carcinogenesis, thus representing a pathological condition where GS overexpression has clinical significance^27^.

To test if oncogenic β-catenin activation could modulate the non-canonical activity of GS, we administered AAV8-TBG-Cre to adult mice carrying one copy of the *Ctnnb1*^lox(ex3)^ allele^28^. The deletion of exon 3 from *Ctnnb1* resulted in the expression of a constitutively active β-catenin in the liver, causing expansion of the GS^+^ zone (Fig. 5f) and consequent accumulation of *N*^5^-methylglutamine in liver tissue (Fig. 5g). Remarkably, while glutamine levels were not significantly altered in the sera of β-catenin-mutant mice (Fig. 5h), *N*^5^-methylglutamine was increased by ~75% compared to Cre^–^ controls (Fig. 5i). These results confirmed that circulating *N*^5^-methylglutamine is a stringent readout for hepatic GS expression, suggesting its possible use as a biomarker for β-catenin-mutant hepatocellular carcinoma (HCC).

### *N*^5^-methylglutamine as a biomarker for GS-expressing HCC

To further test the validity of this hypothesis, we generated a mouse model of liver cancer driven by the combined expression of *Ctnnb1*^lox(ex3)^ and MYC (*Rosa26*^DM.lsl-MYC/DM.lsl-MYC^)^29^, two oncogenic events co-occurring in HCC.

**Fig. 6:**
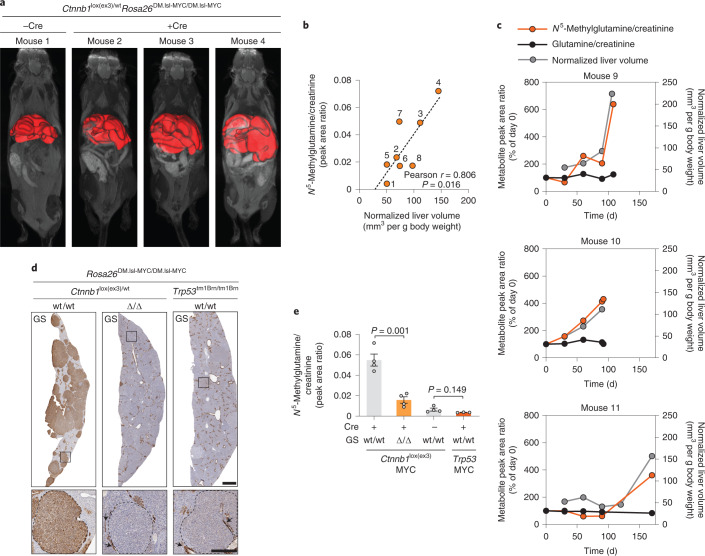
*N*^5^-methylglutamine levels correlate with GS^+^ liver tumor progression. **a**, MRI images of *Cnntb1*^fl(ex3)/wt^*Rosa26*^DM.lsl-MYC/DM.lsl-MYC^ mice scanned 121–156 d after administration of AAV8-TBG-Cre (mice 2, 3 and 4, females). A non-induced mouse (1, female) is shown as a control. Three-dimensional reconstructions of the livers are highlighted in red. **b**, Correlation between the normalized levels of *N*^5^-methylglutamine in the urine of eight mice (1–7, females; 8, male) described in **a** and their normalized liver volumes. Urine samples were collected 24–48 h before the MRI. Data were analyzed by two-tailed Pearson correlation analysis (*n* = 8). **c**, Normalized urine levels of *N*^5^-methylglutamine and glutamine and normalized liver volumes measured in three male mice with the genotype described in **a**. Day 0 corresponds to the day of AAV8-TBG-Cre administration. Individual data points are shown for three mice. **d**, Representative images of IHC staining for GS in liver sections from *Ctnnb1*^lox(ex3)/wt^*Rosa26*^DM.lsl-MYC/DM.lsl-MYC^ male mice with wt/wt or Δ/Δ alleles and *Rosa26*^DM.lsl-MYC/DM.lsl-MYC^*Trp53*^tm1brn/tm1brn^ male mice. Mice were sampled at clinical endpoints between 55 and 164 d after AAV8-TBG-CRE administration; scale bar, 1 mm. The insets show magnifications of tumor regions delineated by dotted lines; scale bar, 250 µm. Arrows indicate normal GS^+^ hepatocytes surrounding central veins. **e**, Normalized urine levels of *N*^5^-methylglutamine from the mice described in **d** (*n* = 4, *Ctnnb1* MYC; *n* = 3, *Trp53* MYC) or in age-matched control male mice administered AAV8-Null-Cre (*n* = 4). Data were analyzed by two-tailed Student’s *t*-test. Bars represent mean ± s.e.m. Source data

The administration of AAV8-TBG-Cre resulted in liver-specific neoplastic lesions that progressed to clinical endpoint with a median survival of 131 and 161 d after induction for male and female mice, respectively. Crucially, we observed tumor-specific high expression of GS (Extended Data Fig. 4j). The ratio between liver volume, measured in live mice by magnetic resonance imaging (MRI), and body weight was used as an index for tumor burden (Fig. 6a). Urine samples were collected before MRI from seven tumor-bearing mice and one age-matched wt/wt control mouse. *N*^5^-methylglutamine was detectable in the urine of wt/wt mice, and its creatinine-normalized levels were increased in all mice with β-catenin/c-MYC-driven tumors, demonstrating a significant positive correlation with tumor burden (Pearson *r* = 0.8 and *P* = 0.016; Fig. 6b). Further, the normalized urine levels of *N*^5^-methylglutamine and glutamine were measured during tumor progression along with liver volumes. The results show that *N*^5^-methylglutamine, but not glutamine, increases synchronously with the tumor burden (Fig. 6c). To ascertain the causal link between GS expression in the tumors and *N*^5^-methylglutamine levels, we generated a line of mice with the *Ctnnb1*^lox(ex3)^, MYC (*Rosa26*^DM.lsl-MYC/DM.lsl-MYC^) and *Glul*^tm3Whla fl^ alleles. The administration of AAV8-TBG-Cre to these mice did not affect the expression of GS in the untransformed liver and resulted in β-catenin/c-MYC-driven tumors deleted for GS (Fig. 6d). The urine and circulating levels of *N*^5^-methylglutamine from these mice were significantly lower than those from mice with GS-expressing tumors (Fig. 6e and Extended Data Fig. 4k). Finally, we found that *N*^5^-methylglutamine urine levels were not increased in mice with GS^–^ p53-null/c-MYC liver tumors or in mice with GS^+^ pancreatic tumors (*Kras*^LSL.G12D/+^; *Trp53*^R172H/+^; *Pdx1*-Cre (KPC))^11^ compared to tumor-free mice (Fig. 6e and Extended Data Fig. 4l), strengthening the validity of this newly discovered GS-derived metabolite as a selective biomarker for β-catenin-mutant liver cancer.

## Discussion

In this study, we demonstrated that GS accepts methylamine as a substrate for the ATP-dependent synthesis of *N*^5^-methylglutamine. The gene encoding GS is one of the most ancient genes found in all forms of life^1^. Hence, it is reasonable to speculate that alongside glutamine synthesis, this previously unreported activity of GS was selected during evolution. The identification of *N*^5^-methylglutamine opens new avenues of research to investigate the relevance of its biosynthesis in the physiopathology of organs with high expression of GS. *N*^5^-methylglutamine is a glutamine analog, and its biological functions could interfere with glutamine transport and catabolism. Indeed, our results suggest that *N*^5^-methylglutamine regulates the hepatic levels of α-ketoglutarate, a product of glutamine degradation with important metabolic and signaling functions^30^. Despite the apparent low affinity of GS toward methylamine observed in vitro with recombinant enzymes and cells, GS activity in the pericentral hepatocytes controls the urine, serum and hepatic concentrations of *N*^5^-methylglutamine, ~10 μM, ~0.8 μM and ~24 μM, respectively (Extended Data Fig. 4m–o).

The non-canonical activity of GS in the liver is fed by the methylamine produced by the microbiota, as demonstrated by inoculating germ-free mice with gut-resident microorganisms. These results place GS in the context of the gut–liver axis, where GS acts as a gatekeeper for methylamine while controling the systemic levels of *N*^5^-methylglutamine. In addition, we showed that cells with high expression of GS derived from brain tumors efficiently synthesize *N*^5^-methylglutamine, demonstrating that the GS-dependent production of *N*^5^-methylglutamine is not unique to specialized hepatocytes. In the brain, GS is highly expressed in astrocytes, and its glutamine-synthesizing activity is essential for glutamatergic neurotransmission, a process where the GS-derived *N*^5^-methylglutamine could play a role.

While the biological effects of *N*^5^-methylglutamine remain to be explored in different physiopathological contexts, we demonstrated that the levels of *N*^5^-methylglutamine are increased in the urine of a β-catenin-mutant genetically engineered mouse model of liver cancer with high GS expression. The levels of *N*^5^-methylglutamine in blood and urine, unlike those of glutamine, are stringent readouts of hepatic GS activity. This finding could lead to the development of a diagnostic tool valuable for GS-expressing WNT/β-catenin-driven HCC, a disease with growing incidence in western society, which currently lacks reliable biomarkers for treatment stratification^31^.

## Methods

### Mouse studies

Animal experiments were either subject to review by the University of Ghent Animal Ethics Committee or were performed in accordance with UK Home Office Regulations (project licenses 70/8645 60/4181, PP6345023 and PP0604995) and subject to review by the Animal Welfare and Ethical Review Board of the University of Glasgow. Mice were housed in ventilated cages with ad libitum food and water access and 12-h light/12-h dark cycles. The temperature of the facility was kept between 19 and 23 °C with 55 ± 10% humidity. *Glul*^tm3Whla fl^ mice^8^ in a C57BL/6 mixed background were kindly provided by M. Mazzone (KU Leuven). *Glul*^tm3Whla fl^ mice were bred in-house and were backcrossed (N5–N12) into a C57BL/6J background, and all other mice were on a mixed C57BL/6J background unless otherwise stated. No statistical calculation was done to determine the sample size. Comparable numbers of male and female *Glul*^tm3Whla fl^ mice received a single injection of AAV8-TBG-Cre virus (2 × 10^11^ genomic copies per mouse) at ~60 d of age, and samples were collected at ~120 d of age unless indicated otherwise. Mice heterozygous for *Ctnnb1*^lox(ex3)^ (ref. ^28^) received a single injection of AAV8-TBG-Cre virus or AAV8-Null-Cre (2 × 10^11^ genomic copies per mouse) at 61–71 d of age. After 4 d, the mice were culled, and tissue and blood were sampled. Male and female mice carrying the *Ctnnb1*^lox(ex3)/wt^ and *Rosa26*^DM.lsl-MYC/DM.lsl-MYC,29^ alleles without or with *Glul*^tm3Whla fl^ alleles received a single injection of AAV8-TBG-Cre virus (6.4 × 10^8^ genomic copies per mouse) at ~80 d of age. Triple mutants were backcrossed into C57BL/6J N10. Male mice backcrossed into C57BL/6J N10 carrying the *Rosa26*^DM.lsl-MYC/DM.lsl-MYC^ and *Trp53*^tm1brn/tm1brn^ alleles received a single injection of AAV8-TBG-Cre virus (5 × 10^10^ genomic copies per mouse) at ~57 d of age^32^. Mice were randomly assigned to experimental groups based on their genotypes, and, with the exception of MRI quantification, the analyses were not performed blindly.

### Germ-free mice

Axenic 8-week-old C57BL6/J mice from the Ghent Germ-Free and Gnotobiotic Mouse Facility at Ghent University were transferred from flexible film isolators (NKP) to positive-pressure isocages (Tecniplast). Colon and cecum content from one 8-week-old C57BL6/J SPF mouse was isolated under anaerobic conditions and homogenized in 5 ml of sterile PBS with 0.1% l-cysteine. The suspension was left for 5 min to let particulates settle, and the supernatant was transferred to a 50-ml Falcon tube and used as donor material. Five germ-free C57BL6/J mice received an oral gavage with 200 µl of SPF donor microbiota suspension, and six germ-free C57BL6/J mice received an oral gavage with sterile PBS with 0.1% l-cysteine. Both groups were housed in positive-pressure isocages for 3 weeks before mice were killed and tissue was collected.

### Stable isotope tracing, methylamine administration and GS inhibition in vivo

Wt/wt and Δ/Δ mice were injected intraperitoneally (IP) with 2 g per kg (body weight) U-^13^C_6_ glucose (CLM-1396, Cambridge Isotopes) or 2 mmol per kg (body weight) ^13^C-methylamine (277630, Sigma-Aldrich), and tissue samples were collected 30 min and 2 h after injections, respectively. Wt/wt mice were IP injected with 200 mg per kg (body weight) U-^13^C_5_-glutamine (CLM-1822, Cambridge Isotopes). Shortly before injection, blood was collected by tail vain puncture and immediately diluted 1:50 in the extraction solution for LC–MS analysis. Thirty minutes after injection, mice were killed, and blood was collected by cardiac puncture and processed as described above. Methylamine (426466, Sigma-Aldrich) was supplemented in the drinking water (0.1% (wt/vol)) to 6- to 9-week-old wt/wt and Δ/Δ mice for 5 months. Water consumption was not affected by methylamine supplementation, and age-matched mice not administered methylamine were used as controls.

Six- to 9-week-old wt/wt mice were injected IP either with NaCl 0.9% (vehicle solution) or 10 mg per kg (body weight) MSO (M5379, Sigma-Aldrich). Blood was collected by tail vain puncture shortly before (0 h) and 2, 4, 8 and 24 h after MSO injection and immediately diluted 1:50 in the extraction solution for LC–MS analysis. Tissue samples were collected for analysis 4 h after MSO administration.

### MALDI imaging

Serial sections of wt/wt and Δ/Δ livers were cut at 10-μm thickness, processed for standard immunohistochemistry (IHC) for GS and OAT or mounted on IntelliSlides (1868957, Bruker) for MALDI imaging. Sections from wt/wt and Δ/Δ livers were paired on each slide. Freeze-dried sections were shipped to the Bruker Daltonics facilities and sprayed with *N*-(1-naphthyl)-ethylenediamine dihydrochloride matrix with a TM-sprayer (HTX Technologies). Data were acquired on a timsTOF fleX instrument (Bruker) in negative Q-TOF ion mode at a 10-µm pixel size. Metabolic compounds were automatically annotated using MetaboScape 2021b (Bruker), and ion distributions were visualized with SCiLS Lab 2021c (Bruker).

### Magnetic resonance imaging and urine collection

At 121–156 d after AAV8-TBG-Cre administration, urine samples were collected from *Cnntb1*^lox(ex3)/wt^*Rosa26*^DM.lsl-MYC/DM.lsl-MYC^ mice and immediately diluted 1:50 in LC–MS extraction solution. Within 24 h of urine collection, MRI images were acquired on a nanoScan PET/MRI (Mediso) using the 35-mm radiofrequency coil. A non-gated T1-weighted gradient echo sequence in the coronal/sagittal plane was acquired in three dimensions with a repetition time of 20 ms, echo time of 3.8 ms and flip angle of 15°. The image matrix is non-isotropic with dimensions of 179 × 512 × 60 and a field of view of 3.58 × 10.24 × 3.00 cm. Standard Fourier transform was used for reconstruction. No postprocessing was performed, and manual segmentation was performed blindly using VivoQuant ver4.0 (Invicro).

### Cell lines

HEK293 and HepG2 cell lines were obtained from ATCC. T16 cells were kindly provided by S. Niclou (Luxembourg Institute of Health). HEK293 and HepG2 cells were routinely cultured with MEM with 1 g liter^–1^ glucose (21090022, Gibco) supplemented with 2 mM glutamine (Gibco), 1% non-essential amino acids (11140035, Gibco), 1 mM pyruvate (S8636, Sigma-Aldrich) and 10% FBS (10270106, Gibco). T16 cells were routinely cultured in MEM (21090022, Gibco) supplemented with 1% non-essential amino acids (11140035, Gibco), 0.65 mM glutamine (A2916801, Gibco), 0.1 mM pyruvate (S8636, Sigma-Aldrich), 1% ITS (1 g liter^–1^ insulin, 550 mg liter^–1^ transferrin, 670 μg liter^–1^ sodium selenite; 41400045, Gibco), 10 ng ml^–1^ epidermal growth factor (EGF; AF-100-15, Peprotech), 10 ng ml^–1^ fibroblast growth factor (FGF; AF-100-18B, Peprotech), 6.8 µg ml^–1^ vitamin B12 (V6629, Sigma-Aldrich), 2 µg ml^–1^ heparin (H3393, Sigma-Aldrich) and 400 mg liter^–1^ AlbuMAX II (11021029, Thermo Fisher Scientific). For the experiments with HEK293 and HepG2 cell lines, cells were seeded in complete MEM, and 15–24 h later, the medium was replaced with Plasmax^33^ supplemented with 2.5% FBS (10270106, Gibco) and 200 mg liter^–1^ AlbuMAX II (11021029, Thermo Fisher Scientific) for an additional 24 h before the experiments. For the experiments, T16 cells were cultured in Plasmax^33^ supplemented with AlbuMAX II (11020029, Thermo Fisher Scientific), ITS, FGF, EGF and heparin at the concentrations used for routine culture. All cell lines were authenticated using the Promega GenePrint 10 kit (B9510, Promega) and tested negative for mycoplasma infection using the MycoAlert mycoplasma detection kit (LT07-318, Lonza).

### CRISPR–Cas9 and overexpression constructs

Non-targeting control sequence and two guide RNAs (gRNAs) against exon 3 of *GLUL* were cloned into LentiCRISPRv2 (refs. ^34,35^) using BsmBI restriction enzyme. For each sequence, 3 × 10^5^ HepG2 or HEK293 cells were transfected with 1 µg of gRNA using jetPrime (Polyplus). Twenty-four hours after transfection, the media were replaced and supplemented with 1 µg ml^–1^ puromycin for selection. Puromycin-resistant cells (500) were seeded in a 25-cm cell culture dish. Individual clones were collected, and GS expression was tested by immunoblotting. The following gRNA target sequences were used: non-targeting control gRNA, 5′-GTAGCGAACGTGTCCGGCGT-3′; *GLUL* gRNA 1, 5′-TCTGTAGGTCCATATTACTG-3′; *GLUL* gRNA 2, 5′-TTCTAGTGGGAATTTCAGAT-3′. The plasmids containing wt, R324A and R324C *GLUL* cDNA were kindly provided by U. Kutay (ETH Zurich)^36^. The *GLUL* coding sequence was subcloned from pcDNA5/FRT/TO/HA into pcDNA3.1 NEO (+) using 5′-BamHI and 3′-EcoRI restriction sites. However, these vectors lack the Kozak sequence upstream of the start codon of *GLUL*, which is required for efficient translation of the full-length GS protein. To add the wt Kozak sequence to the *GLUL* expression cassette, the plasmids were linearized by BamHI and BsrGI digestion, and a repair oligonucleotide was ligated into the plasmid. These repair oligonucleotides contain the sequence for five nucleotides upstream of the *GLUL* start codon (5′-CCACCATG-3′). The sense sequence was

5′-ATCCACCATGACCACCTCAGCAAGTTCCCACTTAAATAAAGGCATCAAGCAGGT-3′, and the antisense sequence was

5′-GTACACCTGCTTGATGCCTTTATTTAAGTGGGAACTTGCTGAGGTGGTCATGGTG-3′. *GLUL* sequences were confirmed by Sanger sequencing.

### Enzymatic assays

Enzymatic assays were performed using a modified protocol described previously^37,38^. Briefly, 2 µg of human recombinant GS obtained from Novus Biologicals (NBP2-52376) or purified GS obtained as described in the next section was added to the reaction mixture consisting of 100 mM imidazole/HCl (pH 7.2; I5513, Sigma-Aldrich), 50 mM glutamate (pH 7.2; G5889, Sigma-Aldrich), 20 mM ATP (pH 7.2; A7699, Sigma-Aldrich) and 20 mM MgCl_2_ (M4880, Sigma-Aldrich). Unless otherwise indicated, 500 µl of reaction mixture was incubated for 2 min at 37 °C, and the reaction was initiated by adding NH_4_Cl (A9434, Sigma-Aldrich), CH_3_NH_2_·HCl (M0505, Sigma-Aldrich), ^15^NH_4_Cl (299251, Sigma-Aldrich) or ^13^C-methylamine (277630, Sigma-Aldrich) at the concentrations indicated in Fig. 4f,g or in the legends of Extended Data Fig. 3a,b. Aliquots (5 µl) of the reaction mixtures were sampled at the times specified in the figure legends, diluted 1:1,000 in LC–MS extraction solution and analyzed by LC–MS. *K*_m_ values were calculated in GraphPad 9.4 (Prism) by using a Michaelis–Menten equation. *N*^2^-Methyl-l-glutamine used in Fig. 2j was synthesized by replacing glutamate with 40 mM *N*-methyl-l-glutamate (ICN15555583, Fisher Scientific) in the reaction mixture described above. The reaction was incubated at 37 **°**C for 2 h, and an aliquot of the mixture was diluted 1:1,000 for LC–MS/MS analysis.

### Expression and purification of human wt GS and R324C mutant

Full-length human *GLUL* wt and R324C mutant were amplified using 5′ primer (5′-TAAGCAGGATCCACCACCTCAGCAAGTTCCCACTTAAATAAAGGC-3′) with BamHI and 3′ primer (5′- TGCTTAGAATTCTTAATTTTTGTACTGGAAGGGCTCATCGCCGG-3′) with EcoRI from pCDNA3.1 GS–HA. Following double digestion and agarose gel purification, *GLUL* fragments were ligated into pRSF-DUET containing 12×His-SUMO, and the sequence was confirmed with Sanger sequencing. Chemically competent *Escherichia coli* BL21(DE3) Rosetta2 pLysS (Novagen) cells were transformed with 12×His-SUMO–GS in pRSF-DUET. Cell cultures were grown in Luria–Bertani medium supplemented with 1 mM MgSO_4_ at 37 °C to an optical density at 600 nm of ~0.8 and induced with 0.35 mM isopropyl β-d-1-thiogalactopyranoside; expression occurred overnight at 16 °C. Cells were collected, centrifuged (600*g*), resuspended in IMAC buffer A (25 mM sodium phosphate, 500 mM NaCl and 50 mM imidazole, pH 7.5) and lysed with a microfluidizer at 10,000 psi. The lysate was cleared by spinning at 19,000 r.p.m. for 45 min at 4 °C, syringe filtered using a 0.45-μm filter and loaded onto a 5-ml His-Trap HP column (GE Life Sciences). The loaded column was washed for 30 column volumes (cv) in IMAC buffer A and eluted with 100% IMAC buffer B (25 mM sodium phosphate, 500 mM NaCl and 350 mM imidazole, pH 7.5) for 5 cv. Fractions were combined and dialyzed against ULP1 cleavage buffer (25 mM Tris, 500 mM NaCl and 5 mM β-mercaptoethanol, pH 8.0) overnight at 4 °C in 3,500 molecular weight cutoff (MWCO) SnakeSkin dialysis tubing (Thermo Fisher) with 5 μM SUMO protease (ULP1). The cleavage reaction was filtered using a 0.45-μm syringe filter and pass backed over a 15-ml His-Trap HP column (GE Life Sciences). The flowthrough was concentrated in a 10,000-MWCO Amicon centrifugal filter unit (Merck Millipore) to a final volume of 3 ml and loaded on a 26/600 Superdex 200 SEC column (GE Life Sciences) preequilibrated in SEC buffer (20 mM HEPES, 300 mM NaCl and 0.5 mM TCEP, pH 7.5). Full-length GS eluted ~0.59 cv, and pure fractions confirmed by SDS–PAGE were concentrated to 67–117 μM using a 10,000-MWCO Amicon centrifugal filter unit. Protein aliquots were stored at −80 °C until use.

### LC–MS metabolomics

Cells were extracted as previously described^33^. Briefly, cells plated in six wells were washed three times with ice-cold PBS and incubated for 5 min at 4 °C with 400 µl of LC–MS extraction solution (20% water, 50% methanol and 30% acetonitrile).

Tissue fragments (20–40 mg) were extracted by using the Precellys Evolution homogenizer (Bertin) and 25 µl of LC–MS extraction solution per mg of tissue. Cell and tissue extracts were centrifuged at 16,000*g* for 10 min at 4 °C, and the supernatant was stored at −74 °C until LC–MS analysis. Compound peak areas obtained for cells were normalized on the total micrograms of proteins determined for each extracted well with a modified Lowry assay^33^.

Metabolites from the biological extracts were injected (5 μl) and separated using a ZIC-pHILIC column (SeQuant; 150 mm × 2.1 mm, 5 μm; Merck) coupled with a ZIC-pHILIC guard column (SeQuant; 20 mm × 2.1 mm) using an Ultimate 3000 HPLC system (Thermo Fisher Scientific). Chromatographic separation was performed using a 15-min linear gradient starting with 20% ammonium carbonate (20 mM, pH 9.2) and 80% acetonitrile, terminating at 20% acetonitrile at a constant flow rate of 200 μl min^–1^. The column temperature was held at 45 °C.

A Q Exactive Orbitrap mass spectrometer (Thermo Fisher Scientific) equipped with electrospray ionization was coupled to the HPLC system for both metabolite profiling and metabolite identification. For profiling, the polarity switching mode was used with a resolution (RES) of 35,000 or 70,000 at 200 *m*/*z* to enable both positive and negative ions to be detected across a mass range of 75 to 1,000 *m*/*z* (automatic gain control (AGC) target of 1 × 10^6^ and maximal injection time (IT) of 250 ms).

Data-dependent fragmentation was performed to aid metabolite identification using a wt/wt liver pooled sample comprised of a mixture of all sample extracts analyzed per experimental batch. The Q Exactive was operated in positive and negative polarity mode separately (35,000 RES, AGC target of 1 × 10^6^ and max IT of 100 ms), and the ten most abundant ions were chosen for fragmentation (minimum AGC target of 1 × 10^3^, AGC target of 1 × 10^5^, max IT of 100 ms, 17,500 RES, stepped normalized collision energy of 25, 60 and 95, isolation width of 1 *m*/*z*, dynamic exclusion of 15 s and charge exclusion of >2) per survey scan.

Data-independent fragmentation was performed to acquire fragmentation spectra of specific metabolites including 5-methylglutamine (positive polarity, *m*/*z* 161.0920). Fragmentation spectra were continuously recorded with the following parameters: 17,500 RES, isolation width of 0.7 *m*/*z*, AGC target of 1 × 10^5^, max IT of 250 ms and stepped normalized collision energy of 25, 60 and 95.

Untargeted metabolomics analysis was performed using Compound Discoverer software (Thermo Scientific v3.2). Retention times were aligned across all data files (maximum shift of 2 min and mass tolerance of 5 ppm). Unknown compound detection (minimum peak intensity of 1 × 10^6^) and grouping of compounds were performed across all samples (mass tolerance of 5 ppm and retention time tolerance of 0.7 min). Missing values were filled using the software’s ‘Fill Gap’ feature (mass tolerance of 5 ppm and signal/noise tolerance of 1.5). Compound identification was assigned by matching the mass and retention time of observed peaks to an in-house library generated using metabolite standards (mass tolerance of 5 ppm and retention time tolerance of 0.5 min) or by matching fragmentation spectra to mzCloud (www.mzcloud.org; precursor and fragment mass tolerance of 10 ppm and match factor threshold of 60).

Targeted metabolomics analysis was performed using Tracefinderv4.1 (Thermo Scientific), and the peak areas of metabolites were determined by using the *m*/*z* of the singly charged ions (extracted ion chromatogram, ±5 ppm) and the retention time from our in-house metabolite library.

*N*^5^-methylglutamine was quantified in the serum and urine samples by a standard addition method. The concentrations of d,l-*N*^5^-methylglutamine indicated in Extended Data Fig. 4h, m–o were obtained by spiking a stock solution of the compound to a solution extracted and pooled from the respective fluid samples (*n* = 4 mice for serum and *n* = 5 mice for urine). The same method was used to quantify *N*^5^-methylglutamine in wt/wt liver samples (*n* = 1) spiked with d,l-*N*^5^-methylglutamine to obtain final concentrations of 0, 1, 5, 10 and 20 µM. To estimate the micromolar concentration of *N*^5^-methylglutamine in liver tissue, we used a ratio of 1 mg of wet tissue per µl. The samples used for the quantification of serum concentrations were analyzed with the Q Exactive operated in positive-selective ion monitoring mode (70,000 RES, AGC target of 2 × 10^5^, max IT of 240 ms and *m*/*z* of 161.0919 ± isolation window of 1 *m*/*z*) using the same chromatographic conditions as above. All other samples were analyzed as described above for biological extracts.

Methylamine was quantified with an LC–MS method adapted from previous reports^39,40^. An aliquot of 25 µl of mouse serum was transferred to an Eppendorf tube, and 5 µl of trichloroacetic acid (20% in water) was added and mixed by vortexing for 30 s. The samples were centrifuged at 12,000*g* for 10 min, and 15 µl of the supernatant was transferred to a new tube and supplemented with 22.5 µl of borate buffer (0.5 M, pH 11) and 12.5 µl of tosyl chloride (10 mg ml^–1^ in acetonitrile). The mixture was mixed by vortexing for 5 s and incubated for 2 h at 50 °C. The samples were cooled down at room temperature and analyzed by LC–MS. A selected reaction monitoring mode was used to detect derivatized methylamine on an Altis QQQ mass spectrometer equipped with a Vanquish LC system (Thermo Fisher Scientific). Chromatography was performed on an Acquity HSS T3 column (Waters; 150 mm × 2.1 mm, 1.8 μm). The mobile phase consisted of solvent A (water with 0.1% formic acid) and solvent B (acetonitrile with 0.1% formic acid). Separation of metabolites was performed with the following gradient: 0 min 20% B, 8 min 95% B and 10 min 20% B at a constant flow rate of 0.3 ml min^–1^. The injection volume was 5 µl. Three transitions were optimized using a standard of derivatized methylamine from the positive precursor ion (*m*/*z* 185.9) to product ions (*m*/*z* 64.8, 90.8 and *m*/*z* 154.9). The total cycle time was 0.8 s, and Q1 RES was (full-width at half-maximum) 0.7 and Q3 RES was (full-width at half-maximum) 1.2. For each transition, the collision energy applied was optimized to generate the greatest possible signal intensity and using the calibrated RF values. The optimized source parameters were a spray voltage of 3,500 V, sheath gas of 35, aux gas of 7, ion transfer tube temperature of 325 °C and vaporizer temperature of 275 °C. Data acquisition was performed using Xcalibur 4.1 (Thermo Scientific) software, and quantification was performed using Tracefinderv4.1 (Thermo Scientific).

### Ammonia measurement

Ammonia concentration was measured in frozen sera or in blood collected from the tail vein of mice and immediately analyzed with the blood ammonia meter PocketChem BA PA-4140 (Arkray).

### Immunohistochemistry

Tissue samples were fixed in a 10% solution of neutral buffered formalin overnight (16–24 h) and transferred to 70% ethanol. Paraffin-embedded tissue blocks were cut into 5-μm sections and stained with hematoxylin and eosin or with the following antibodies: GS (1:800; HPA007316, Sigma-Aldrich) and OAT (1:200; ab137679, Abcam). IHC images were visualized with an Aperio ImageScope v12.4 (Leica Biosystems).

### Immunoblotting

Cells were washed twice with ice-cold PBS, and proteins were extracted with RIPA buffer (20-188, EMD Millipore) containing protease and phosphatase inhibitors (A32961, Thermo Fisher Scientific). Protein amounts were quantified with a standard bicinchoninic acid assay (A32961, Pierce). Tissues were extracted with 25 µl of RIPA buffer per mg of wet weight. Tissue fragments were homogenized with the Precellys Evolution homogenizer (Bertin), and 20–80 μg of protein extract was loaded in 9.5% acrylamide gels for electrophoresis and blotted onto nitrocellulose membranes. PageRuler prestained protein ladder (26616, Thermo Fisher Scientific) was used as a reference for the protein molecular weight. Membranes were incubated overnight with the following primary antibodies: GS (1:1,000; 610517, BD Bioscience), OAT (1:1,000; ab137679, Abcam), β-actin (1:1,000; ab8229, Abcam), β-tubulin (1:2,000; T5201, Sigma-Aldrich) and vinculin (1:1,000; V9131, Sigma-Aldrich). The secondary antibodies anti-rabbit horseradish peroxidase (1:1,000; 7074, Cell Signaling Technology), anti-mouse IRDye 800CW (1:2,500; 926-32212, Licor) and anti-goat IRDye 680CW (1:2,500; 926-68074, Licor) were used, and membranes were imaged with an Odyssey infrared scanner and visualized with Image Studio Lite 5.2 (Licor) or imaged with Clarity Western ECL substrate (1705061, Bio-Rad) and a Chemidoc MP imager (Bio-Rad) and visualized with Chemidoc Image Lab 6.0 (Bio-Rad). Scanned images of uncropped membranes are shown in the Source Data.

### Reporting summary

Further information on research design is available in the Nature Research Reporting Summary linked to this article.

## Online content

Any methods, additional references, Nature Research reporting summaries, source data, extended data, supplementary information, acknowledgements, peer review information; details of author contributions and competing interests; and statements of data and code availability are available at 10.1038/s41589-022-01154-9.

## Supplementary information

Reporting summary

## Data Availability

All the data supporting the findings of this study are available within the article. Source data files that support the findings of this study are stored at the Cancer Research UK Beatson Institute and are available from the corresponding author upon reasonable request. Requests for unique biological materials can be made to the corresponding author. Source data are provided with this paper.

## Source data

Source Data Fig. 1 Statistical source data.
Source Data Fig. 1 Unprocessed western blots.
Source Data Fig. 2 Statistical source data.
Source Data Fig. 3 Statistical source data.
Source Data Fig. 4 Statistical source data.
Source Data Fig. 4 Unprocessed western blots.
Source Data Fig. 5 Statistical source data.
Source Data Fig. 6 Statistical source data.
Source Data Extended Data Fig. 1 Statistical source data.
Source Data Extended Data Fig. 1 Unprocessed western blots.
Source Data Extended Data Fig. 3 Statistical source data.
Source Data Extended Data Fig. 3 Unprocessed gels.
Source Data Extended Data Fig. 4 Statistical source data.
