# Supplementary material for: Hepatic glutamine synthetase controls N5-methylglutamine in homeostasis and cancer
Source: Nat Chem Biol. 2022 Oct 24;19(3):292–300. doi: 10.1038/s41589-022-01154-9 (PMC9974483; doi:10.1038/s41589-022-01154-9)
Supplement: Supplementary file 1 — Reporting summary [file 41589_2022_1154_MOESM1_ESM.pdf]

nature portfolio

Corresponding author(s): Saverio Tardito

Last updated by author(s): Aug 18, 2022

Reporting Summary

Nature Portfolio wishes to improve the reproducibility of the work that we publish. This form provides structure for consistency and transparency in reporting. For further information on Nature Portfolio policies, see our Editorial Policies and the Editorial Policy Checklist.

Statistics

For all statistical analyses, confirm that the following items are present in the figure legend, table legend, main text, or Methods section.

n/a	Confirmed	
☐	☒	The exact sample size (*n*) for each experimental group/condition, given as a discrete number and unit of measurement
☐	☒	A statement on whether measurements were taken from distinct samples or whether the same sample was measured repeatedly
☐	☒	The statistical test(s) used AND whether they are one- or two-sided<br>*Only common tests should be described solely by name; describe more complex techniques in the Methods section.*
☐	☒	A description of all covariates tested
☐	☒	A description of any assumptions or corrections, such as tests of normality and adjustment for multiple comparisons
☐	☒	A full description of the statistical parameters including central tendency (e.g. means) or other basic estimates (e.g. regression coefficient) AND variation (e.g. standard deviation) or associated estimates of uncertainty (e.g. confidence intervals)
☐	☒	For null hypothesis testing, the test statistic (e.g. *F*, *t*, *r*) with confidence intervals, effect sizes, degrees of freedom and *P* value noted<br>*Give P values as exact values whenever suitable.*
☒	☐	For Bayesian analysis, information on the choice of priors and Markov chain Monte Carlo settings
☒	☐	For hierarchical and complex designs, identification of the appropriate level for tests and full reporting of outcomes
☐	☒	Estimates of effect sizes (e.g. Cohen's *d*, Pearson's *r*), indicating how they were calculated

Our web collection on statistics for biologists contains articles on many of the points above.

Software and code

Policy information about availability of computer code

Data collection	MALDI imaging data were acquired on a timsTOF fleX instrument (Bruker) in negative Q-TOF ion mode at 10 µm pixel size.<br>MRI images were acquired on a nanoScan PET/MRI (Mediso, Budapest, Hungary).<br>Immunoblots were acquired with an Odyssey scanner (Licor) or Chemidoc MP imager (Biorad).<br>Mass Spectrometry data were acquired with Thermo Scientific Xcalibur Version 4.1 and subsequent versions.
Data analysis	Targeted metabolomics analysis was performed using Tracefinderv4.1 (Thermo Scientific).<br>Untargeted metabolomics analysis was performed using Compound Discoverer (Thermo Scientific v3.2).<br>MALDI imaging analysis was performed using MetaboScape 2021b (Bruker) and ion distributions visualized with SCiLS Lab 2021c (Bruker).<br>MRI imaging analysis was performed with VivoQuant ver4.0 (Invicro).<br>Immunoblots images were analyses with Image Studio Lite 5.2 (Licor) and Chemidoc image lab 6.0 (Biorad).<br>Mass spectrometry data were analyzed with Thermo Scientific Xcalibur Version 4.1 and subsequent versions.<br>Immunohistochemistry images were visualized with Aperio ImageScope v12.4 (Leica Biosystems).<br>The glutamine synthetatse protein structure (PDB 2QC8) was visualized with UCSF Chimera v1.15.

For manuscripts utilizing custom algorithms or software that are central to the research but not yet described in published literature, software must be made available to editors and reviewers. We strongly encourage code deposition in a community repository (e.g. GitHub). See the Nature Portfolio guidelines for submitting code & software for further information.

Data

Policy information about availability of data

All manuscripts must include a data availability statement. This statement should provide the following information, where applicable:

- Accession codes, unique identifiers, or web links for publicly available datasets
- A description of any restrictions on data availability
- For clinical datasets or third party data, please ensure that the statement adheres to our policy

All the data supporting the findings of this study are available within the article, and the supplementary information files. Source data file that support the findings of this study are stored at the Cancer Research UK Beatson Institute and are available from the corresponding author upon reasonable request. Requests for unique biological materials can be made to the corresponding author.

Field-specific reporting

Please select the one below that is the best fit for your research. If you are not sure, read the appropriate sections before making your selection.

☒ Life sciences ☐ Behavioural & social sciences ☐ Ecological, evolutionary & environmental sciences

For a reference copy of the document with all sections, see nature.com/documents/nr-reporting-summary-flat.pdf

Life sciences study design

All studies must disclose on these points even when the disclosure is negative.

Sample size	For animal experiments no statistic calculation was performed to predetermine the sample size. Sample size was chosen based on pilot experiments with small number of animals (n=2-4), or based on the outcome of similar experiments previously carried out at the same institution with similar murine models. For all other experiments no sample size calculation was performed, and sample sizes were chosen based on experience gained from previous studies.
Data exclusions	No data were excluded.
Replication	Multiple independent experiments were performed for cell culture-based studies as indicated in the figure legends. Tissue sections or fragments from at least 3 animals per genotype or condition were used for analysis, unless mentioned otherwise.
Randomization	Mice were randomly assigned to experimental groups taking into account their genotype and sex. For in vitro experiments group allocation was not relevant.
Blinding	Researchers were not blinded to genotype of animals reaching clinical endpoints at different stages for animal welfare reasons. The investigator(s) were blinded to genotype or treatment for MRI data analysis.

Reporting for specific materials, systems and methods

We require information from authors about some types of materials, experimental systems and methods used in many studies. Here, indicate whether each material, system or method listed is relevant to your study. If you are not sure if a list item applies to your research, read the appropriate section before selecting a response.

Materials & experimental systems

n/a	Involved in the study	
☐	☒	Antibodies
☐	☒	Eukaryotic cell lines
☒	☐	Palaeontology and archaeology
☐	☒	Animals and other organisms
☒	☐	Human research participants
☒	☐	Clinical data
☒	☐	Dual use research of concern

Methods

n/a	Involved in the study	
☒	☐	ChIP-seq
☒	☐	Flow cytometry
☒	☐	MRI-based neuroimaging

Antibodies

Antibodies used	Glutamine synthetase (HPA007316, Sigma-Aldrich), Glutamine synthetase (610517, BD Bioscience), Ornithine aminotransferase (ab137679, Abcam), β-actin (ab8229, Abcam), β-tubulin (T5201, Sigma-Aldrich), Vinculin (V9131, Sigma-Aldrich), anti-Rabbit-HRP (1:1000; 7074, Cell Signaling Technology), anti-Mouse-IRDye 800CW (1:2500; 926-32212, Licor), Anti-Goat-IRDye 680CW (1:2500; 926-68074, Licor).

Validation	The glutamine synthtetase antibodies were validated with cell and mouse models genetically engineered for GLUL in this study Fig 1c,d and 3h.<br>All other antibodies used were validated for the species and applications used in this study (WB and IHC) as stated at the respective manufacturer's websites.

Eukaryotic cell lines

Policy information about cell lines

Cell line source(s)	HEK293 and HepG2 cell lines were obtained from ATCC. T16 cells are not commercially available and were previously characterized in Golebiewska et al. 2020;140(6):919-949. doi: 10.1007/s00401-020-02226-7.
Authentication	All cell lines were authenticated using Multiplex PCR-based STR analysis (Promega GenePrint 10 Kit, B9510, Promega)
Mycoplasma contamination	All cell lines tested negative for mycoplasma infection using the MycoAlert Mycoplasma Detection Kit (LT07-318, Lonza).
Commonly misidentified lines<br>(See ICLAC register)	No commonly misidentified cell line was used in this study.

Animals and other organisms

Policy information about studies involving animals; ARRIVE guidelines recommended for reporting animal research

Laboratory animals	Mice with the following alleles Glul tm3Whla; Ctnnb1 lox(ex3); Rosa26DM.lsl-MYC; Trp53 tm1brn; K-rasLSL.G12D; Trp53R172H; Pdx-1-Cre were bred to obtain the allelic combiations indicated in the manuscript. Mice of both sexes aged eight weeks to 12 months, on a mixed background or backcrossed (N5-12) into a C57BL/6J background were used in this study. Mice were housed in University of Glasgow (UK) facilities, with the exception of germ-free mice housed at the Germ-free and Gnotobiotic Mouse facility at Ghent University, Belgium.
Wild animals	This study did not use wild animals.
Field-collected samples	This study did not use field collected samples.
Ethics oversight	Animal experiments were either subject to review by the University of Ghent Animal Ethics Committee or were performed in accordance with UK Home Office Regulations (project licences 70/8645 60/4181, PP6345023, PP0604995) and subject to review by the Animal Welfare and Ethical Review Board of the University of Glasgow.

Note that full information on the approval of the study protocol must also be provided in the manuscript.
