# Supplementary figures and images for: Hepatic glutamine synthetase controls N5-methylglutamine in homeostasis and cancer
Source: Nat Chem Biol. 2022 Oct 24;19(3):292–300. doi: 10.1038/s41589-022-01154-9 (PMC9974483; doi:10.1038/s41589-022-01154-9)

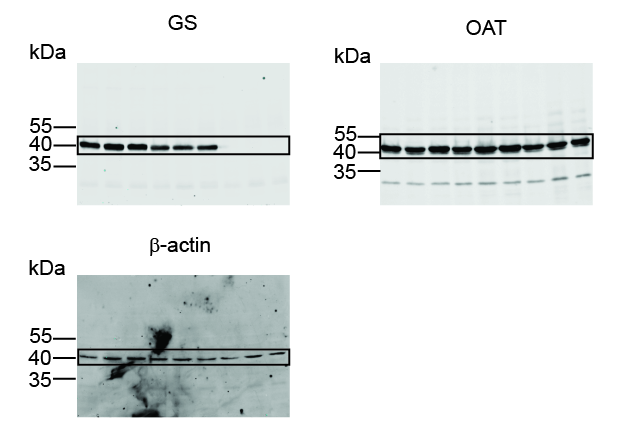

Supplement: Source Data Fig. 1 — Unprocessed western blots. [file 41589_2022_1154_MOESM3_ESM.tif]

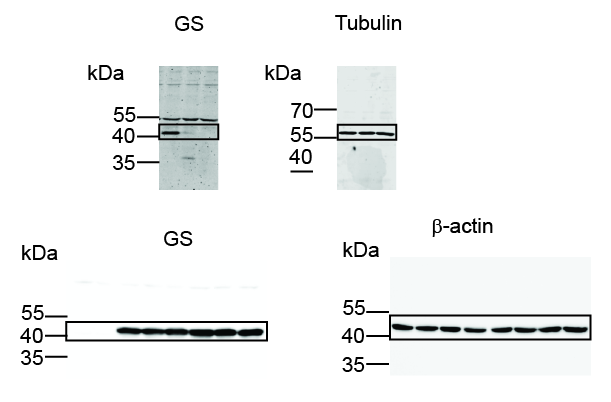

Supplement: Source Data Fig. 4 — Unprocessed western blots. [file 41589_2022_1154_MOESM7_ESM.tif]

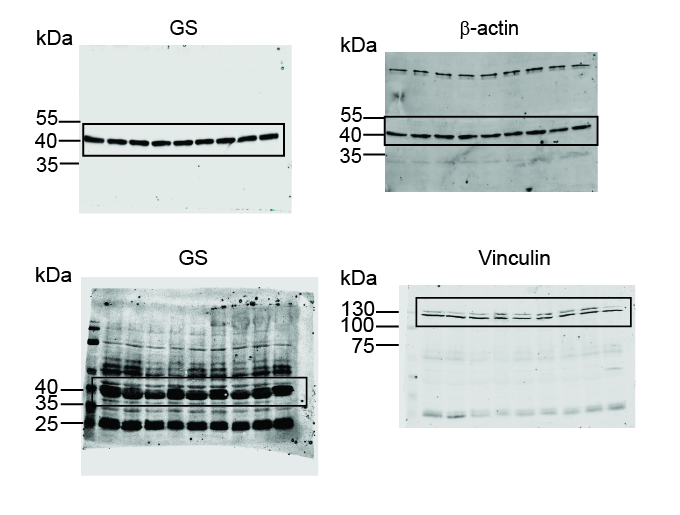

Supplement: Source Data Extended Data Fig. 1 — Unprocessed western blots. [file 41589_2022_1154_MOESM11_ESM.tif]

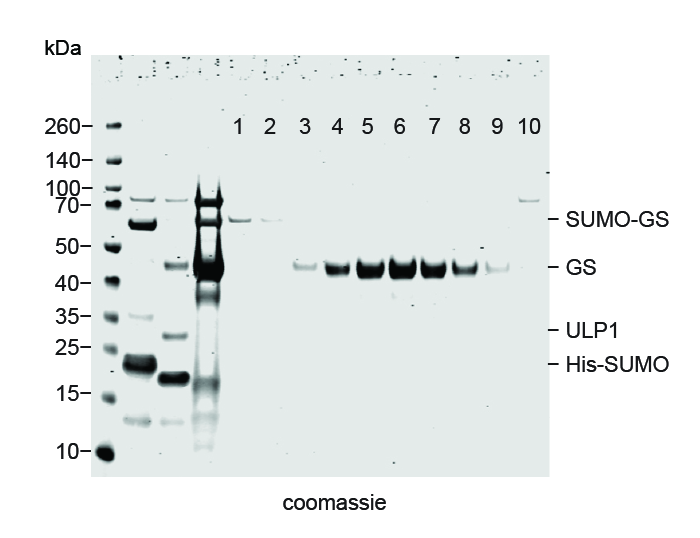

Supplement: Source Data Extended Data Fig. 3 — Unprocessed gels. [file 41589_2022_1154_MOESM13_ESM.tif]
